# Heparanase Promotes Tumor Growth and Liver Metastasis of Colorectal Cancer Cells by Activating the p38/MMP1 Axis

**DOI:** 10.3389/fonc.2019.00216

**Published:** 2019-04-02

**Authors:** Xue Liu, Zhi-hang Zhou, Wen Li, Shi-kun Zhang, Jing Li, Ming-Ju Zhou, Jin-Wen Song

**Affiliations:** ^1^Department of Pathology, College of Basic Medicine, Jining Medical University, Jining, China; ^2^Department of Gastroenterology, The Second Affiliated Hospital of Chongqing Medical University, Chongqing, China; ^3^Department of Tissue Engineering, Beijing Institute of Transfusion Medicine, Beijing, China; ^4^Treatment and Research Center for Infectious Diseases, Beijing 302 Hospital, Beijing, China

**Keywords:** colorectal cancer, heparanase, liver metastasis, MMP1, extracellular matrix

## Abstract

Heparanase (HPSE), the only known mammalian endoglycosidase responsible for heparan sulfate cleavage, is a multi-faceted protein affecting multiple malignant behaviors in cancer cells. In this study, we examined the expression of HPSE in different colorectal cancer (CRC) cell lines. Gene manipulation was applied to reveal the effect of HPSE on proliferation, invasion, and metastasis of CRC. Knockdown of HPSE resulted in decreased cell proliferation *in vitro*, whereas overexpression of HPSE resulted in the opposite phenomenon. Consistently, *in vivo* data showed that knockdown of HPSE suppressed tumor growth of CRC. Furthermore, knockdown of HPSE inhibited invasion and liver metastasis *in vitro* and *in vivo*. RNA-sequencing analysis was performed upon knockdown of HPSE, and several pathways were identified that are closely associated with invasion and metastasis. In addition, HPSE is positively correlated with MMP1 expression in CRC, and HPSE regulates MMP1 expression via p38 MAPK signaling pathway. In conclusion, our data demonstrate that HPSE knockdown attenuated tumor growth and liver metastasis in CRC, implying that HPSE might serve as a potential therapeutic target in the treatment of CRC.

## Introduction

Colorectal cancer (CRC) is the third most common diagnosed cancer and the fourth leading cause of cancer-related mortality worldwide. Moreover, tumor invasion and metastasis are the main causes of mortality in CRC patients (1, 2). The liver is the most common site of CRC metastasis and more than 50% of CRC patients will develop metastatic liver disease (3, 4). Therefore, identification of the mechanisms involved in the invasion and metastasis of CRC is urgently needed.

The first process in the invasion and metastasis of tumor cells is the breakage of the tissue barrier formed by the basement membrane and extracellular matrix (ECM). Heparanase (HPSE), the only recognized mammalian endo-β-D-glucuronidase, is responsible for the cleavage of heparan sulfate (HS) chains of heparan sulfate proteoglycans (HSPGs) in the ECM as well as on the tumor cell surface (5). HPSE is first synthesized as a latent ~65 kDa precursor protein and then post-translationally cleaved into ~8 and ~50 kDa subunits that non-covalently associate to form the active HPSE heterodimer (6). HSPGs are important components of the ECM, and the activity of HPSE could lead to the degradation of the ECM, which facilitates tumor cell invasion (7). In addition, HS serves as a storage depot for many cytokines, including VEGF, bFGF and HGF (8, 9). Cleavage of HS by HPSE leads to the release of bioactive cytokines, which promotes tumor growth and angiogenesis. The upregulation of HPSE has been implicated in many types of cancers, including CRC (7, 10–13). Both HPSE mRNA and protein are practically undetectable in morphologically normal colon epithelium, but are induced during colon carcinogenesis (14–16). The presence of HPSE in CRC correlates with higher TNM stage, higher vascular infiltration, and higher lymph vessel infiltration (15). In addition, HPSE expression is frequently detected in the invasive front of CRC (15). Furthermore, the 5-year survival rate is higher for patients with negative HPSE expression, and significant correlations were reported between HPSE expression and liver metastasis (10). Importantly, HPSE-transfected RPMI 4788 CRC cells showed increased invasiveness in invasion chamber assays, and the HPSE inhibitor SF-4 suppressed the invasion of RPMI 4788 cells (15). Although the clinical significance of HPSE is well documented, the mechanisms of HPSE-mediated CRC invasion and metastasis need to be further explored.

In this study, CRISPR/Cas9 technology was used to manipulate HPSE expression in CRC cells. We showed that knockdown of HPSE suppresses CRC cell proliferation, invasion, and liver metastasis. RNA-sequencing revealed that genes and pathways involved in remodeling of ECM were attenuated upon HPSE knockdown. These finding suggest that HPSE might be an attractive target for the treatment of CRC.

## Materials and Methods

### Cell Lines and Cell Culture

The human colorectal cancer cell lines SW480, SW620, LoVo, HT-29, and HCT116, were obtained from the American Type Culture Collection (ATCC, Manassas, VA, USA). Cells were cultured in DMEM (Gibco, Carlsbad, CA, USA) supplemented with 10% fetal calf serum (FCS) (Gibco), 100 U/mL penicillin, and 100 U/mL streptomycin (Gibco). All cells were cultured in a humidified 5% CO_2_ incubator at 37°C.

### Lentiviral Constructs and Cell Infection

HPSE overexpression was induced with a lentiviral vector system, as previously described (13). Briefly, SW620 cells were infected with HPSE lentiviral activation particles or control lentiviral activation particles (Santa Cruz Biotechnology, Santa Cruz, CA, USA). The lentiviral activation particles contain the following SAM activation elements: a deactivated Cas9 nuclease (D10A and N863) fused to the transactivation domain VP64, and a blasticidin resistance gene; an MS2-p65-HSF1 fusion protein, and a hygromycin resistance gene; a target-specific 20nt guide RNA, and a puromycin resistance gene. Stable infected cells were selected with 2 μg/mL puromycin, 400 μg/mL hygromycin B and 5 μg/mL blasticidin S HCl. For convenience, these two cell lines are referred to as SW620-HPSE and SW620-Con, respectively.

For construction of the HPSE knockdown cell line, SW480 cells were infected with Lenti-CAS9-puro (GeneChem Co. Ltd. Shanghai, China) and selected with 2 μg/mL puromycin. Then, cells stably expressing SW480-Cas9 were infected with two lentivirus-sgRNA-EGFP vectors targeting HPSE and a control lentivirus-sgRNA-EGFP, respectively. The sgRNA sequences are listed in Table 1. Exon 4 and Exon 3 of HPSE gene were targeted by KD1 and KD2, respectively. Stable HPSE knockdown cells are referred to as SW480-KD1, SW480-KD2, whereas control cells were named SW480-NC.

**Table 1:** The sgRNAs sequences.

**Number**	**sgRNA (5–3′)**
NC	CGCTTCCGCGGCCCGTTCAA
KD1	ACGGTTGGAATGGCCCTACC
KD2	TTCTCCAAAGCTTCGTACCT

### RNA Extraction and Real-Time Reverse Transcription-PCR

Total cellular RNA was isolated with TRIzol reagent (Invitrogen, Carlsbad, CA, USA) according to the manufacturer's instructions and quantified using a UV spectrophotometer. RNA was then reverse transcribed to cDNA using PrimerScript^TM^ RT master mix (Takara, Dalian, China). mRNA expression was examined by real-time reverse transcription PCR (RT-PCR) using SYBR Green Mix with a CFX96 real-time PCR system (Bio-rad, Richmond, CA, USA). Each reaction was performed in triplicate. The results were normalized to human β-actin mRNA expression. The primers used in this study are listed in Table S1.

### Western Blot Analysis

Proteins were extracted from samples using radio immunoprecipitation assay (RIPA) buffer (Beyotime, Shanghai, China) according to the manufacture's protocol. After gel electrophoresis, PVDF membranes were blocked in 5% milk/PBS-T for 2 h followed by overnight incubation at 4°C with antibodies against HPSE (Proteintech, Chicago, IL, USA; 1:1000, 66226-1-Ig), phosphor-p38 (Cell Signaling, Beverly, MA, USA; 1:2000, 4511), p38 (Cell Signaling; 1:2000, 8690), MMP1 (Proteintech; 1:1000, 10371-2-AP), PCOLCE (OriGene Tech, Rockville, MD, USA; 0.5 μg/mL, TA337676), MMP10 (Abcam, Cambridge, MA, USA; 1:1000, ab199688), CEACAM6 (Abcam; 1:500, ab78029) and β-actin (Sigma- Aldrich, St Louis, MO, USA; 1:5000, A5316). After washes and incubation with respective horseradish peroxidase-conjugated secondary antibodies for 2 h, protein bands were visualized using the SuperSignal West Pico maximum sensitivity substrate (Pierce, Rockford, IL, USA).

### Immunofluorescence Staining

Cells were seeded into 24-well plates containing glass coverslips on the bottom of the wells. Cells were fixed with 4% paraformaldehyde for 30 min, permeabilized with Triton X-100 for 10 min, and blocked with 2.5% bovine serum albumin for 1 h. Cells were then incubated with the anti-HPSE antibody (Proteintech; 1:200, 66226-1-Ig) for 1 h at room temperature, followed by incubation with Alexa Fluor 488-conjugated secondary antibody for 1 h. The cell nucleus was stained with DAPI for 5 min. Images were captured with an inverted fluorescence microscope (PerkinElmer, Norwalk, CT, USA).

### Cell Proliferation Assay

Cell proliferation was determined with the CCK-8 assay. Cells were incubated in 10% CCK-8 (Beyotime), which was diluted in normal culture medium for 1 h at 37°C. The absorbance was measured at 450 nm using a SpectraMax M5 plate reader (Molecular Devices, Sunnyvale, CA, USA).

### Invasion Assays

Invasion assays were performed using 8 μm Transwell chambers coated with Matrigel (BD Biosciences, Bedford, MA, USA). Briefly, cells were suspended in 100 μL serum-free medium and seeded into the upper chamber. The lower chamber was filled with 600 μL DMEM medium supplemented with 10% FCS. After 24 h of incubation, invaded cells were stained with 0.5% crystal violet (Beyotime) and examined by bright field microscopy (Leica, Wetzlar, Germany).

### Animal Experiments

For tumorigenicity assay, 1 × 10^6^ cells were injected subcutaneously into the back of BALB/c nude mice. After 3 weeks, mice were sacrificed; tumor xenografts were harvested, weighted, and fixed in formalin.

For the *in vivo* liver metastasis assay, SW480 cells (2 × 10^6^) were suspended in PBS and then injected into the spleen of 6–8 weeks old male BALB/c nude mice. Five weeks after intrasplenic injection, mice were sacrificed, and spleen and livers specimens were fixed in formalin. Sections (5-μm thickness) of the liver were made at 10 different layers to cover the entire organ and stained with hematoxylin and eosin (H&E). Metastatic foci were counted under microscopy in a double-blinded manner. All experimental procedures and protocols were approved by the Institutional Animal Care and Use Committee.

### Immunohistochemical Analysis and Scoring

Immunohistochemistry was performed as previously described. Briefly, following deparaffinization and rehydration, the sections were boiled in 10 mmol/L citrate buffer (pH 6.0) for 15 min in a microwave oven. The sections were then incubated with anti-HPSE (Proteintech; 1:100, 66226-1-Ig) or anti-Ki67 (MXB Biotech, Fujian, China; 1:100, MAB-0672) antibodies overnight at 4°C. Sections were washed for 2 h in TBST and then incubated with secondary antibody (DAKO, Carpinteria, CA, USA; P0447) at a dilution of 1:100 in TBST. Finally, the sections were visualized using diaminobenzidine solution (DAKO). The results were verified by two pathologists independently.

### RNA Sequencing and Data Analysis

Three independent experiments were performed for each group to obtain biological replicates. RNA was extracted and sequenced by CapitalBio Corporation (Beijing, China). The NEB Next Ultra RNA Library Prep Kit for Illumina (NEB, Beverly, MA, USA) was used to construct libraries for sequencing. The final libraries were quantified using the KAPA Library Quantification kit (KAPA Biosystems, South Africa) and Bioanalyzer 2100 (Agilent, Palo Alto, CA, USA); libraries were then sequenced on a Hiseq 2500 platform (Illumina, San Diego, USA).

Differentially expressed genes (DEGs) were assessed using Cuffdiff, with a false discovery rate correction for multiple testing. DEGs were considered significant when the log2 signal ratio was ≥ 1.0 or ≤ −1.0 with a *p*-value < 0.05 between comparisons. By searching the ENSEMBL, NCBI, Uniprot, GO, and KEGG databases, BLAST (Basic Local Alignment Search Tool) alignment was performed to determine the functional annotation of DEGs. The best matches were selected to annotate the DEGs. Then, these DEGs were grouped and analyzed using Gene Ontology (GO) enrichment analysis. And, these DEGs were grouped into gene pathways using the pathway enrichment analysis with the following databases: KEGG, BioCyc, Reactome, and Panther. Finally, the protein-protein interaction analysis was conducted using STRING database and visualized using Cytoscape software (Cytoscape Consortium, San Diego, CA, USA).

### Statistical Analysis

Statistical analyses were performed using Graphpad Prism 5.0 (Graphpad, San Diego, CA, USA) using Student's *t*-test. Data are presented as the mean ± standard deviation except for where noted. *P* < 0.05 was considered statistically significant.

## Results

### HPSE Expression in CRC Cell Lines

**Figure 1:**
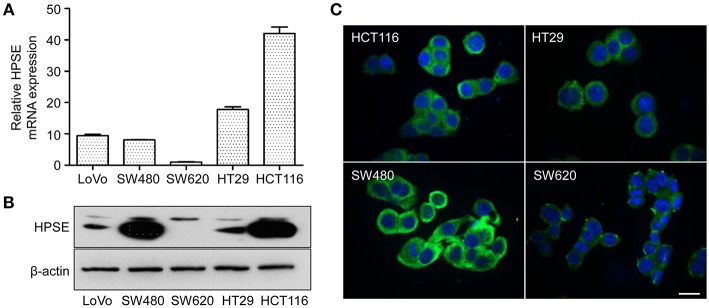
HPSE expression in CRC cells. **(A)** Quantification of HPSE mRNA expression in different CRC cells. The expression of HPSE was normalized to β-actin. Results are shown as the fold change of CRC cells relative to SW620 cells. Results are representative of three independent experiments and are presented as the mean ± SEM. **(B)** Western blot analysis of HPSE expression in whole-cell lysates of CRC cells. SW480 and HCT116 cells exhibited higher expression of HPSE compared to the other cell lines. **(C)** Immunofluorescence staining demonstrated that HPSE was mainly localized in the cytoplasm of CRC cells. Green indicates HPSE and blue indicates DAPI. Scale bar, 20 μm.

The expression of HPSE at the mRNA and protein level was evaluated in five CRC cell lines by qRT-PCR and western blotting, respectively. As shown in Figures 1A,B, SW480, and HCT116 cells express higher levels of HPSE mRNA and protein compared to that in other cells. The lowest expression of HPSE was found in SW620 cells. Immunofluorescence staining revealed that HPSE is located primarily in the cytoplasm of CRC cells (Figure 1C). Since SW480 and SW620 cells were isolated from the same patient, they were selected as the “host” cell to evaluate the functional properties of HPSE in colorectal cancer cells.

### HPSE Promotes Proliferation of CRC Cells *in vitro*

**Figure 2:**
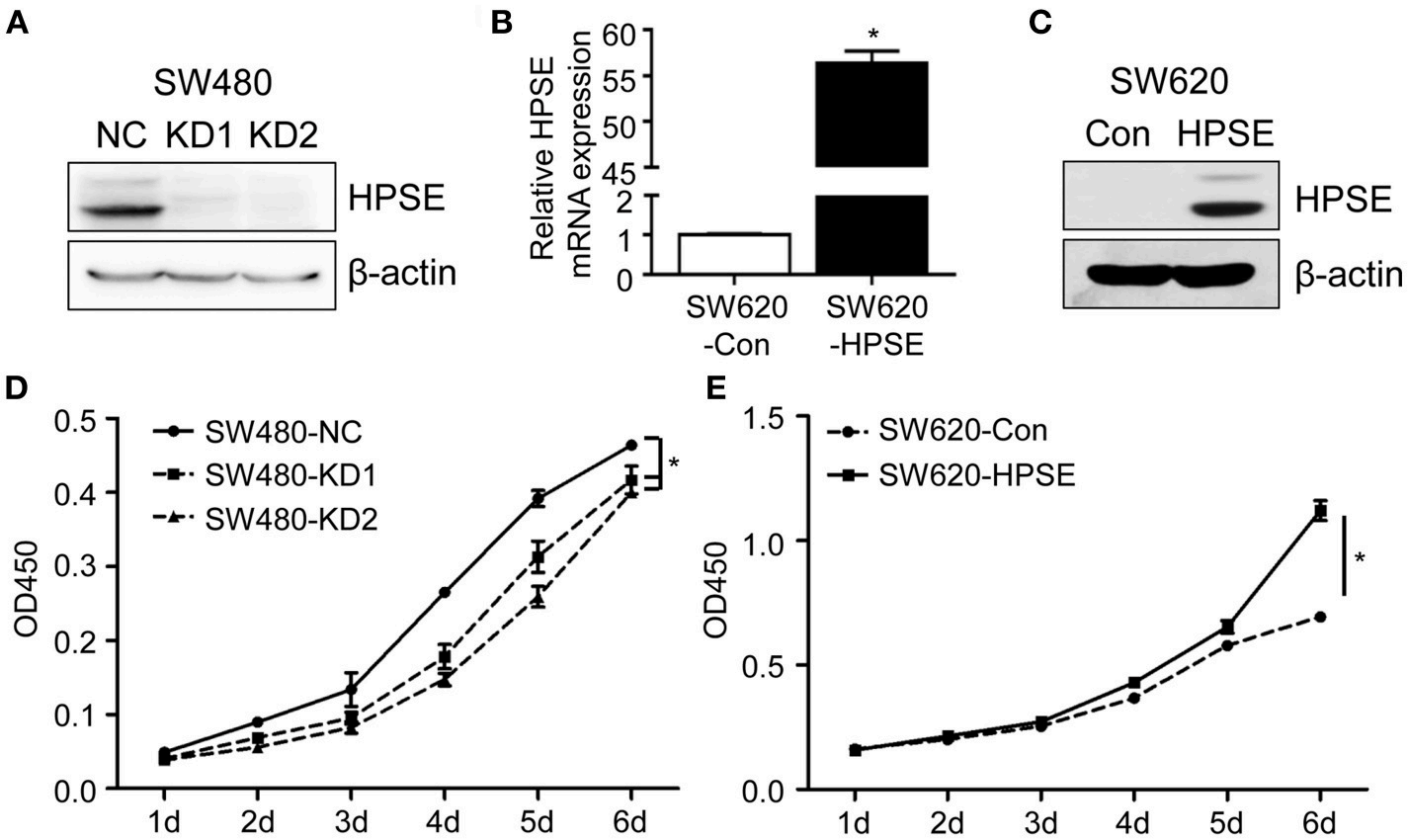
HPSE promotes the proliferation of CRC cells *in vitro*. **(A)** Crispr-Cas9 technology was used to knockdown HPSE expression in SW480 cells and the expression of HPSE was determined by western blotting. **(B,C)** SW620 cells were infected HPSE lentiviral activation particles and HPSE expression was determined by **(B)** qRT-PCR and **(C)** western blotting. The proliferation of **(D)** SW480 cells and **(E)** SW620 cells was determined by the CCK-8 assay (*n* = 3). The OD450 value was assessed at 1, 2, 3, 4, 5, and 6 days respectively. ^*^p < 0.001.

The effect of HPSE on proliferation was reported in other malignancies, but it remains to be investigated in CRC cells. To this end, two gRNAs targeting different regions of the HPSE gene were designed and we transfected the gRNA-Cas9 expression vectors into SW480 cells to generate HPSE knockdown cells (Figure 2A), while SW620 cells were stably transfected with HPSE-expressing particles (Figures 2B,C). Using the CCK-8 assay, we found that HPSE overexpression resulted in significantly increased cell proliferation; in contrast, HPSE knockdown markedly decreased the proliferation of CRC cells (Figures 2D,E).

### HPSE Accelerates Tumor Growth of CRC Cells *in vivo*

**Figure 3:**
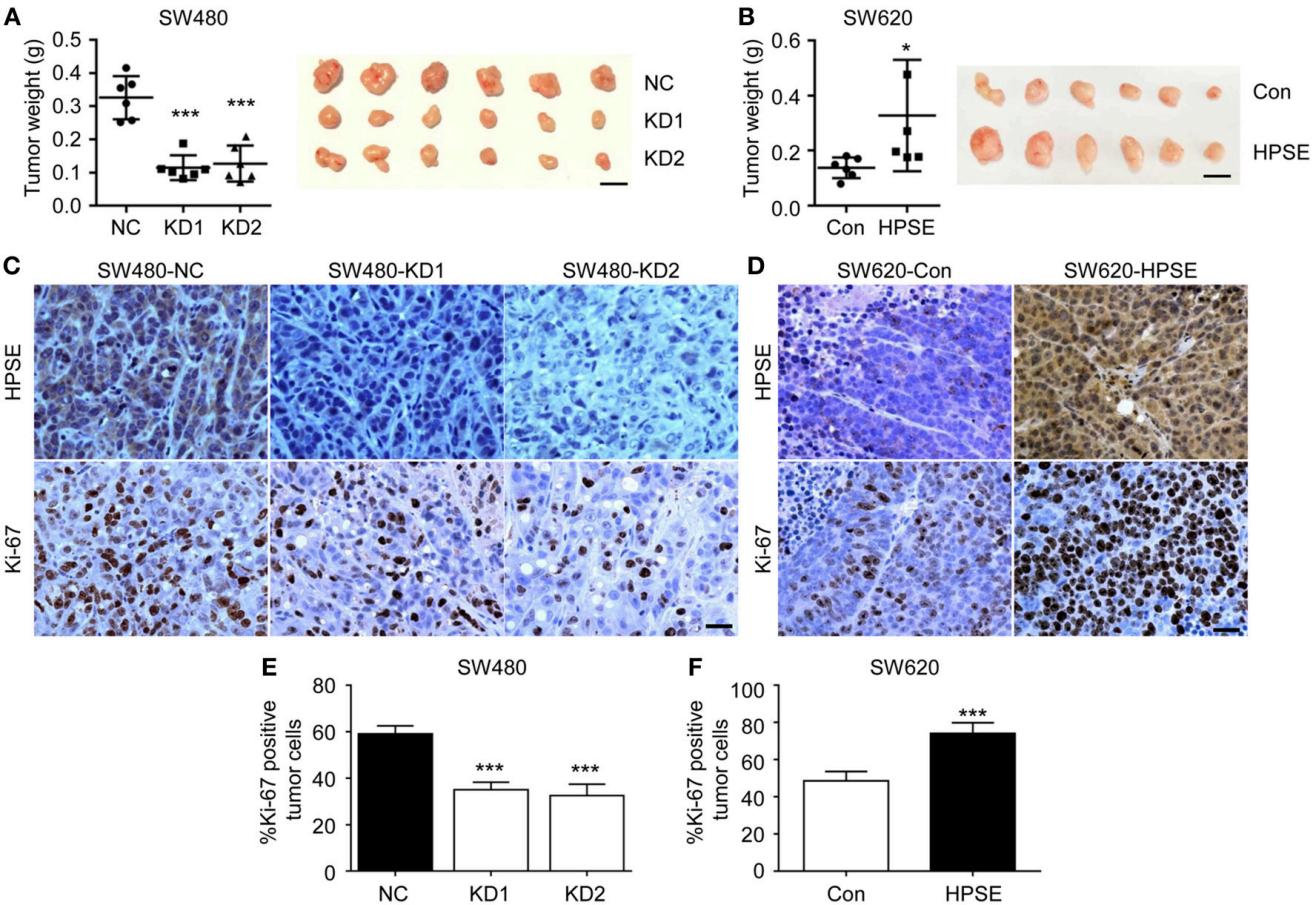
HPSE promotes the proliferation of CRC cells *in vivo*. Cells were injected subcutaneously into the back of BALB/c nude mice. Mice were sacrificed at 3 weeks after transplantation. The xenografts were excised and weighted. **(A)** Knockdown of HPSE expression inhibited tumorigenicity of SW480 cells in BALB/c nude mice (*n* = 6). The weight of tumors originating from SW480-NC is larger than that arising from SW480-KD1 and SW480-KD2 cells (left panel). A representative photograph of tumor size is shown (right panel). **(B)** Overexpression of HPSE in SW620 cells promoted the growth of mouse xenograft tumors (*n* = 6). Scale bar, 1 cm. Immunohistochemistry analysis of HPSE and Ki-67 expression in xenografts originating from **(C)** SW480 and **(D)** SW620 cells. Scale bar, 100 μm. Quantification of tumor cell proliferation in **(E)** SW480 and **(F)** SW620 xenografts using Ki-67 staining (*n* = 6). Data are expressed as Ki-67 positive tumor cells as percentage of total tumor cells. ^*^p < 0.05, ^***^p < 0.001.

To evaluate the role of HPSE in the proliferation of CRC *in vivo*, CRC cells were subcutaneously injected into the flank region of BALB/c nude mice and xenografts were harvested at 3 weeks after transplantation. As shown in Figure 3A, the tumor weight of the SW480 HPSE-knockdown group was much smaller than that in the control group (0.114 ± 0.038 g, 0.127 ± 0.054 g vs. 0.326 ± 0.065 g, *p* < 0.001). In contrast, tumors formed by SW620-HPSE cells were larger than that in SW620-Con cells (0.328 ± 0.202 g vs. 0.1376 ± 0.037 g) (Figure 3B). As Ki67 is frequently used to assess proliferation in human cancer cells, immunohistochemistry analysis was performed to determine Ki67 and HPSE expression in tumors derived from HPSE overexpression and knockdown cells. Representative pictures of Ki67 staining are shown in Figures 3C,D. Compared with SW480-NC group, Ki-67 quantification revealed a significant reduction of tumor cell proliferation in HPSE-knockdown group (59.14 ± 3.43% vs. 35.13 ± 3.14%, 32.50 ± 4.90%, *p* < 0.001) (Figure 3E). Conversely, higher tumor cell proliferation was observed in SW620-HPSE group compared to SW620-Con group (74.16 ± 5.74% vs. 48.59 ± 5.00, *p* < 0.001) (Figure 3F). These data indicate that HPSE promotes the proliferation of CRC cells *in vivo*.

### Knockdown of HPSE Suppresses the Invasion and Metastasis of CRC Cells

**Figure 4:**
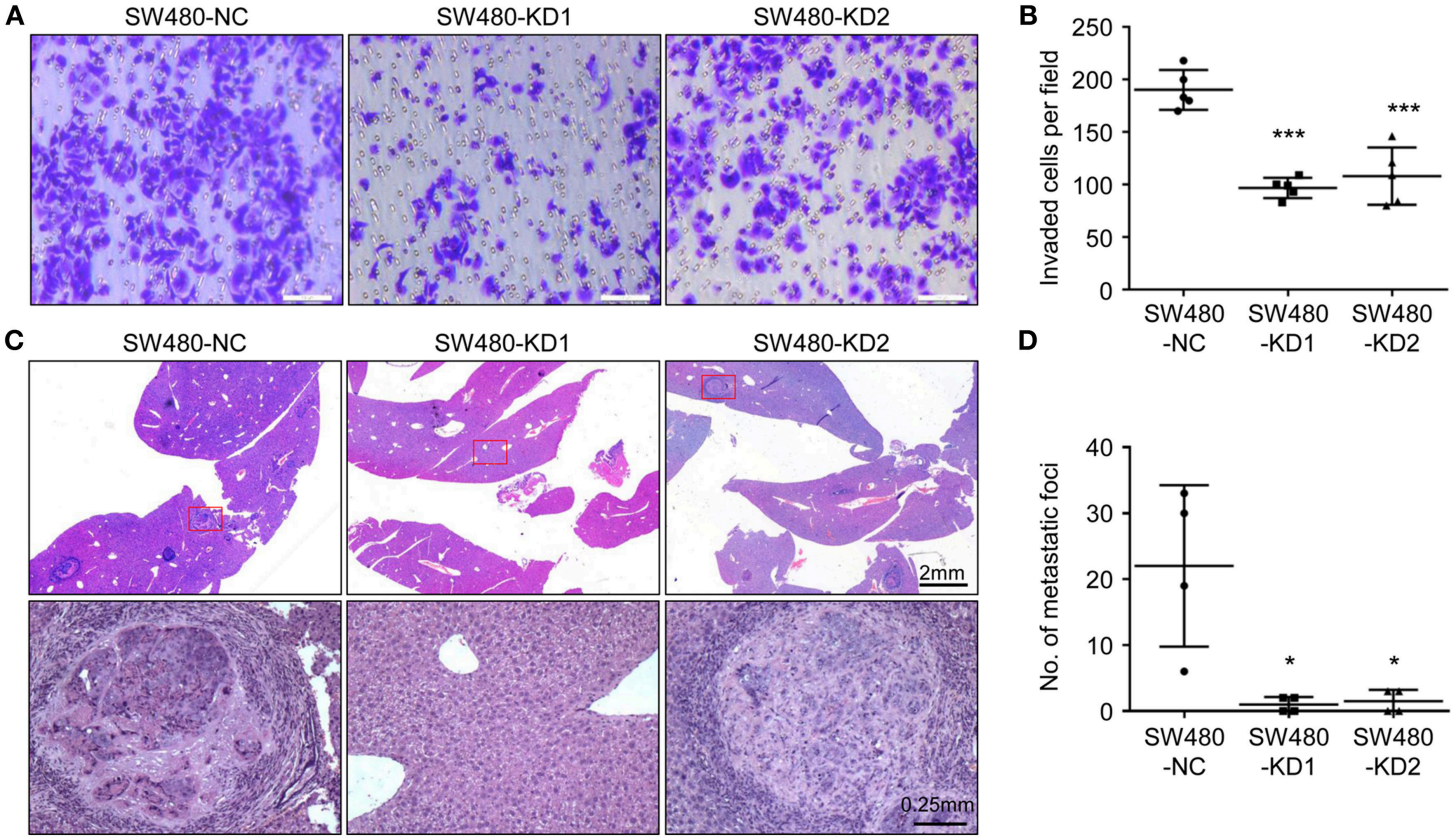
Knockdown of HPSE inhibits CRC cells invasion *in vitro* and liver metastasis *in vivo*. **(A)** An invasion assay was performed in a Matrigel-coated transwell chamber; representative images of invasion assay are shown. **(B)** The invasive ability of SW480 cells was inhibited by HPSE knockdown (*n* = 5). **(C)** SW480 cells were injected into the spleens of BALB/c mice. After 6 weeks, the mice were sacrificed, and their livers were fixed. Microscopic liver metastases were detected by H&E staining. **(D)** The metastatic foci were counted (*n* = 4). HPSE knockdown in SW480 cells resulted in fewer metastatic foci in the liver. ^*^p < 0.05, ^***^p < 0.001.

To explore the effect of HPSE on the invasion of CRC cells, an invasion assay was used to determine the invasiveness of SW480 cells after HPSE knockdown. After 24 h, the cells on the lower surface of the chamber were fixed, stained, and examined under a microscope. Knockdown of HPSE was found to significantly suppress the invasive ability of SW480 cells (Figures 4A,B). To further investigate the pro-metastatic activity of HPSE, SW480 cells were injected into the spleen of 6- week-old male BALB/c nude mice. After 5 weeks, the mice were sacrificed, and their livers were fixed. Metastatic foci in the livers were counted. Our results showed that liver metastasis was significantly inhibited by HPSE knockdown (Figures 4C,D). Combining the two HPSE knockdown groups, metastasis was found in the livers of 50% (4/8) of the mice. In contrast, metastatic foci were found in all livers of the control group. These results suggest that HPSE is critical for tumor invasion and metastasis in CRC.

### Identification of DEGs in HPSE-Knockdown SW480 Cells

**Figure 5:**
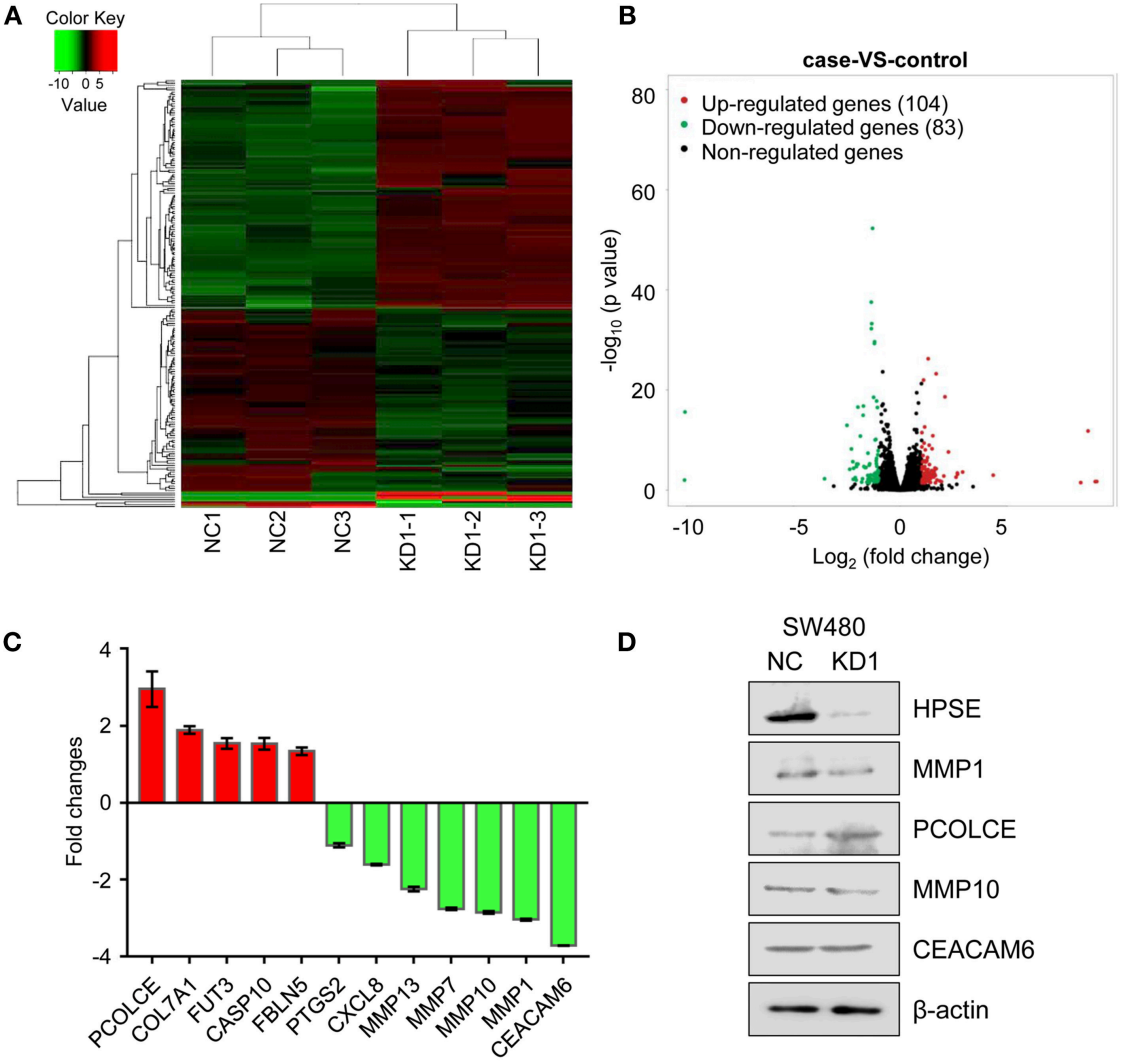
Differentially expressed genes across all samples. **(A)** Hierarchical clustering of differentially expressed mRNA between SW480-KD1 and SW480-NC cells. The relative levels of differentially expressed genes are depicted on the color scale. Red indicates increased relative expression and green indicates decreased relative expression. **(B)** Distribution of the differentially expressed genes is shown as a volcano plot. The detected genes are presented using log2 (fold change) on the x-axis and -log10 (*p*-value) on the y-axis. **(C)** Twelve differentially expressed genes were selected and validated using qRT-PCR. Results are representative of three independent experiments and are presented as the mean ± SEM. **(D)** The protein levels of HPSE, MMP1, PCOLCE, MMP10, and CEACAM6 were also validated by Western blot analysis. β-actin was used as internal control.

To better understand the role of HPSE in CRC cell invasion and metastasis, we took advantage of next generation RNA sequencing technology to analyze mRNA transcriptome differences between SW480-KD1 and SW480-NC cells. The data have been deposited under GEO accession number GSE126504. We identified a total of 104 genes that were significantly upregulated (Table S2) and a total of 83 genes that were significantly downregulated (Table S3) in SW480-KD1. To obtain a global view of these DEGs, hierarchical clustering was constructed (Figure 5A). Additionally, the degree of expression change of these DEGs between the two groups is shown as a volcano plot in Figure 5B. To further validate the reliability of the RNA-seq results, twelve DEGs were selected for qRT-PCR validation, including *PCOLCE, COL7A1, FUT3, CASP10, FBLN5, PTGS2, CXCL8, MMP13, MMP7, MMP10, MMP1*, and *CEACAM6* (Figure 5C). The qRT-PCR results are in accordance with RNA-seq data. Western blot analysis was also used to validate some DEGs, including MMP1, PCOLCE, MMP10, and CEACAM6 (Figure 5D). Except for CEACAM6, the protein levels of MMP1, PCOLCE, and MMP10 are in accordance with RNA-seq data.

**Figure 6:**
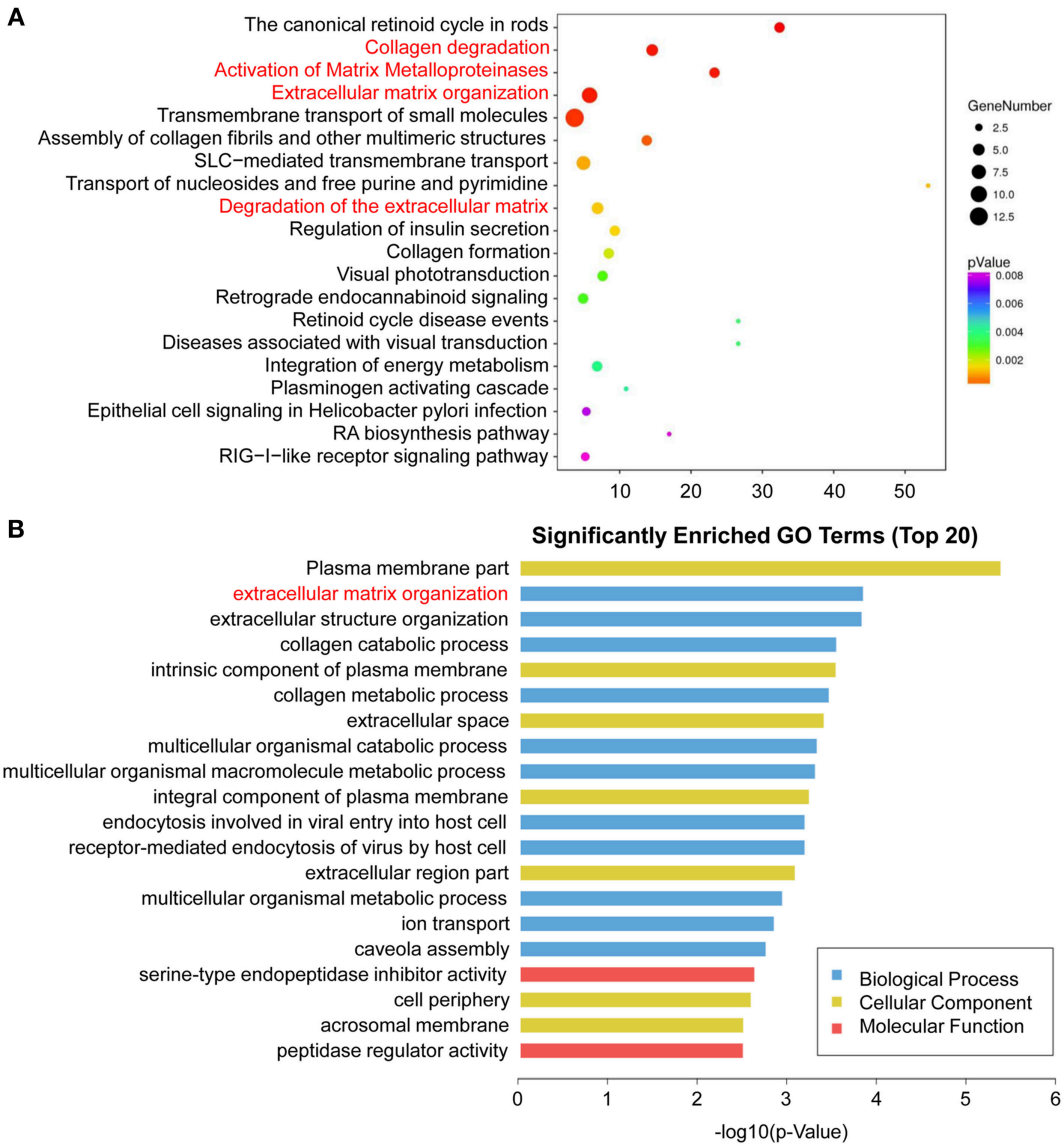
Enriched pathway statistics and GO functional classification of the DEGs. **(A)** Scatter plot of enriched pathway statistics. These differentially expressed genes were grouped into gene pathways using the pathway enrichment analysis with the databases of KEGG, BioCyc, Reactome, and Panther. The color and size of the dots represent the range of the *p*-value and the number of DEGs mapped to the indicated pathways, respectively. Top 20 enriched pathways are shown. **(B)** Top 20 significantly enriched GO terms. The DEGs are summarized in three main categories: biological process (blue), molecular function (red), and cellular component (yellow). The x-axis indicates -log10 (*p*-value) and the y-axis indicates different GO terms.

To identify signaling pathways involved in HPSE-knockdown cells, we mapped the ENSEMBL, NCBI, Uniprot, GO, and KEGG databases; the top 20 enriched pathways are shown in Figure 6A and Table S4. DEGs were highly clustered in several pathways, such as “collagen degradation,” “activation of matrix metalloproteinases.” “Extracellular matrix organization” and “degradation of the extracellular matrix,” suggesting that HPSE may perform its function through the regulation of genes involved in the remodeling of the extracellular matrix.

GO analysis was then performed to group genes with similar function and associations. The top 20 enriched GO terms are shown in Figure 6B and Table S5. In the cellular component category, the DEGs were mostly enriched in plasma membrane part. In the molecular function category, genes associated with serine-type endopeptidase inhibitor activity were enriched. Notably, in the biological process analysis, genes involved in extracellular matrix organization were significantly enriched, including *MMP1, MMP7, MMP10, MMP13, COLA71*, and *FBLN5*.

### HPSE Is Positively Correlated With MMP1 in CRC

**Figure 7:**
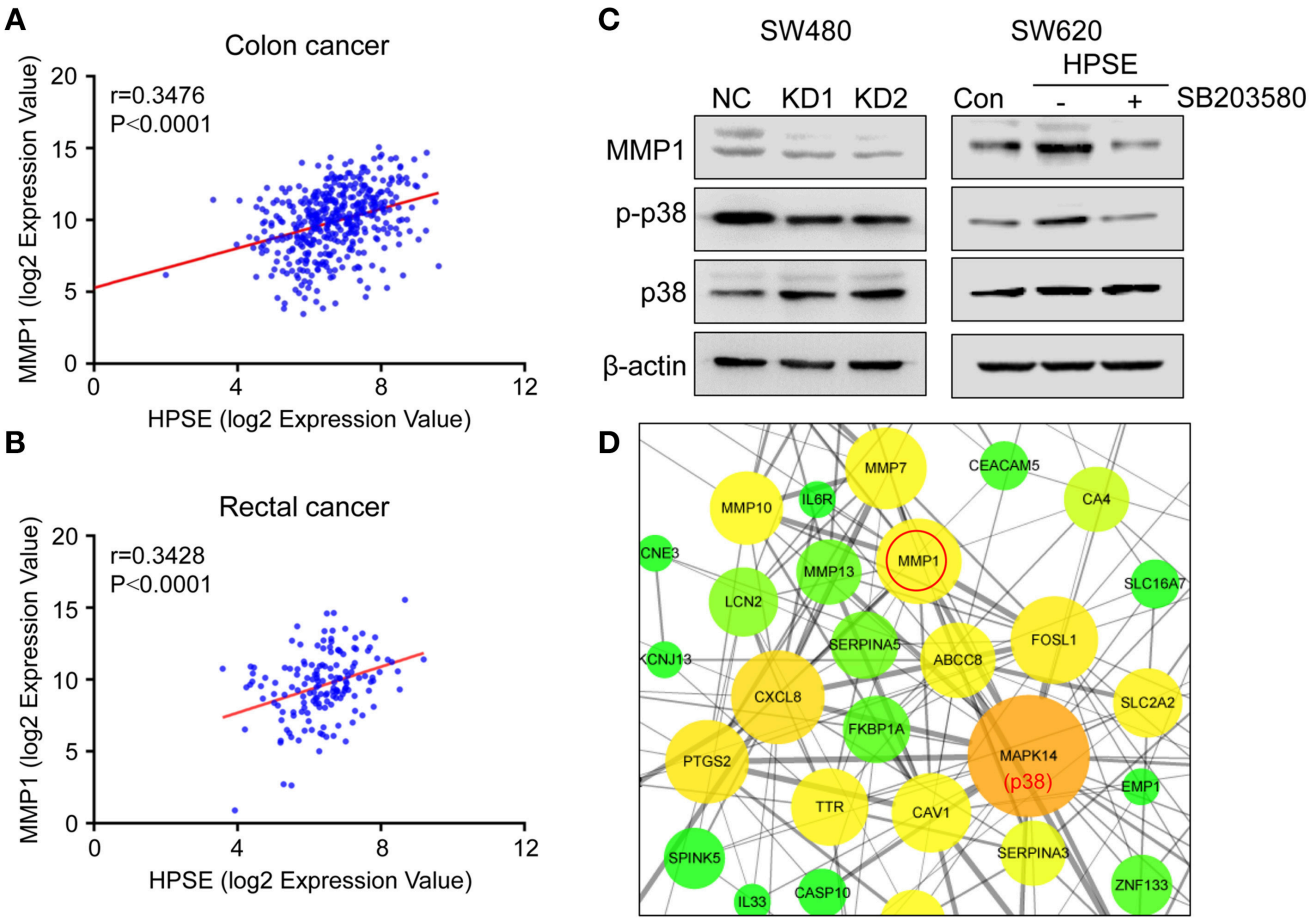
Correlation between the expression of HPSE and MMP1 in colon cancer and rectal cancer. **(A,B)** Positive correlations between the transcriptional levels of HPSE and MMP1 in **(A)** colon cancer and **(B)** rectal cancer. These analyses were performed by analyzing colon and rectal cancer data from TCGA. The correlation between gene expression levels was analyzed by the Pearson correlation test. **(C)** Western blot analysis of MMP1, p38, and p-p38 expression upon knockdown of HPSE (left panel) and overexpression of HPSE (right panel). SW620-HPSE cells were treated with or without p38 pathway inhibitor (SB203580, 10 μM). **(D)** The interactions of the DEGs identified from RNA-seq analysis were extracted from STRING database and visualized using Cytoscape software. The nodes represent proteins and edges represent pairwise interactions. The size of the nodes is proportional to the number of connections established with other genes. The color, from green to red, is used to measure betweenness centrality (BC). The BC quantifies how drastically a gene influences the structure of the whole network. p38 has the largest number of neighboring proteins and MMP1 is tightly linked to p38.

Knockdown of HPSE is accompanied by downregulation of *MMP1, MMP7, MMP10*, and *MMP13*, all of which are directly involved in the degradation of extracellular matrix and facilitate tumor cell invasion. We thus further analyzed the data from TCGA and showed that *HPSE* is positively correlated with the expression of *MMP1* in colon cancer (*r* = 0.3476, *p* < 0.0001) and rectal cancer (*r* = 0.3428, *p* < 0.0001) (Figures 7A,B), but not *MMP7, MMP10*, and *MMP13* (Figure S1).

It has been shown that MMP1 is regulated by the p38 MAPK signaling pathway in colorectal cancer (17). Our results also showed that knockdown of HPSE decreased the expression of MMP1 and the phosphorylation of p38. By contrast, HPSE overexpression led to increased levels of MMP1 and phosphorylation of p38, and the increase was abolished by the treatment of SB203580 (a p38 pathway inhibitor) (Figure 7C). In addition, Cytoscape software was used to analyze protein-protein interaction of these differentially expressed genes. As shown in Figure 7D, MAPK14 (p38) was most enriched with 26 proteins connected, of which MMP1 was one of the proteins having the highest score (score = 882) (Table S6). Collectively, these data indicate that HPSE might regulate MMP1 expression via the p38 MAPK signaling pathway.

## Discussion

The liver is the most common site of CRC metastasis because the majority of intestinal mesenteric drainage enters the hepatic portal venous system (18). High expression of HPSE has been reported in CRC and is correlated with poor prognosis and liver metastasis (10, 15). However, it is still not clear whether HPSE is directly involved in CRC cell invasion and metastasis. Here, we demonstrated that the proliferation, invasion and liver metastasis of CRC cells are inhibited by HPSE knockdown.

HPSE is a highly versatile protein affecting multiple events in tumor progression, including cell adhesion, invasion and angiogenesis. Strategies targeting HPSE have also been developed, including neutralizing antibody, heparan sulfate mimetics, and siRNA (19). CRISPR-Cas9 is a new gene editing technique with high target-specificity, and there is great interest in evaluating its potential for human gene therapy (20). In this study, two gRNAs targeting HPSE were shown to efficiently knockdown HPSE expression (Figure 2A). Several groups have reported that inhibition of HPSE expression by small interference RNA resulted in decreased proliferation and invasion in other malignancies (21–23). Consistent with those results, we also demonstrate that knockdown of HPSE inhibits proliferation and invasion of SW480 cells. In contrast, overexpression of HPSE in SW620 cells resulted in increased proliferation (Figures 2,3). Furthermore, Doniner et al. also reported that overexpression of HPSE in HT29 CRC cells promote xenografts growth (16). Of particular interest, our data demonstrated that knockdown of HPSE attenuated CRC liver metastasis in a mouse model of liver metastasis (Figure 4). To the best of our knowledge, this is the first study to apply CRISPR-Cas9 technology to knockdown of HPSE expression and provide direct evidence of the role of HPSE in liver metastasis of CRC.

Since the mechanisms involved in HPSE-mediated invasion and metastasis have not yet been elucidated, we utilized RNA-seq technology to profile differentially expressed genes and pathways in HPSE-knockdown CRC cells. A total of 187 genes were identified, of which 104 genes were upregulated and 83 downregulated (Figure 5). Interestingly, several genes associated with cancer cells invasion were downregulated upon HPSE knockdown, including *MMP1, MMP7, MMP10, MMP13*, and *CEACAM6* (Figure 5C). Decreased MMP1 and MMP10 protein levels were also observed in HPSE knockdown cells, but not for CEACAM6 (Figure 5D). In addition, PCOLCE was upregulated in HPSE knockdown cells and PCOLCE might have an MMP inhibitory activity (24). Of note, we showed that *HPSE* is positively correlated with *MMP1* expression by analyzing TCGA database (Figures 7A,B). MMP1 is a collagenase that degrades ECM, especially type I, II and III collagens. In CRC, MMP1 expression correlates with advanced stage and poor prognosis, and CRC invasion and migration correlated with increased MMP1 expression (25). Previously, Zetser et al. showed that the levels of p38 phosphorylation were increased upon HPSE overexpression in MDA-MB-453 and HEK293 cells. In this study, we also showed that overexpression of HPSE lead to increased p38 phosphorylation and HPSE knockdown attenuated the p38 phosphorylation in CRC (Figure 7C). In addition, MMP1 has been shown to be tightly regulated by p38 MAPK signaling pathway (17, 26). In our study, inhibition of p38 activity by SB203580 markedly inhibited the induction of MMP1 in HPSE-overexpression cells (Figure 7C, right panel). Moreover, strong interactions between p38 and MMP1 demonstrated by protein-protein interaction analysis (Table S6). Notably, MMP9 has been reported to be regulated by HPSE in myeloma cells (27), but our RNA-seq results did not show difference in MMP9 expression. Taken together, these results suggested that HPSE might regulate MMP1 expression by p38 MAPK signaling pathway.

Additionally, we performed GO analysis, demonstrating that genes associated with “extracellular matrix organization” in the biological process category were enriched (Figure 6B). We further analyzed these DEGs by pathway analysis. Notably, these DEGs are highly enriched in pathway involved in ECM remodeling, including “collagen degradation,” “activation of matrix metalloproteinases,” “extracellular matrix organization” and “degradation of the extracellular matrix,” further suggesting the importance of HPSE in CRC invasion and metastasis (Figure 6A).

In conclusion, our data indicate that knockdown of HPSE can efficiently inhibit the proliferation, invasion and liver metastasis of CRC cells. Furthermore, RNA-seq analysis revealed that HPSE is tightly linked to the pathways involved in ECM remodeling, and therefore contributes to the invasion and metastasis of CRC. These findings demonstrate that HPSE plays a critical role in the regulation of malignant behavior of CRC cells and suggests that HPSE might be an attractive anti-cancer target in CRC.

## Data Availability

Publicly available datasets were analyzed in this study. This data can be found here: http://www.cbioportal.org.

## Ethics Statement

This study was carried out in accordance with the recommendations of the Medical Animal Care and Welfare Committee of Jining Medical University. The protocol was approved by the Medical Animal Care and Welfare Committee of Jining Medical University.

## Author Contributions

ZZ and J-WS conception, design, writing and review of the manuscript. XL, WL, JL, and M-JZ acquisition of data. ZZ, SZ, and J-WS analysis and interpretation of data.

### Conflict of Interest Statement

The authors declare that the research was conducted in the absence of any commercial or financial relationships that could be construed as a potential conflict of interest.
