# Supplementary material for: Heparanase Promotes Tumor Growth and Liver Metastasis of Colorectal Cancer Cells by Activating the p38/MMP1 Axis
Source: Front Oncol. 2019 Apr 2;9:216. doi: 10.3389/fonc.2019.00216 (PMC6454005; doi:10.3389/fonc.2019.00216)
Supplement: Supplementary file 1 [file Table_1.DOCX]

**Supplementary Table 1. The primers used in the present study.**

| Primer's name | sequence | Product length |
| --- | --- | --- |
| HPSE-F | TACCTTCATTGCACAAACACTG | 88 |
| HPSE-R | ACTTGGTGACATTATGGAGGTT |  |
| β-actin-F | CCTGTACGCCAACACAGTGC | 211 |
| β-actin-R | ATACTCCTGCTTGCTGATCC |  |
| PCOLCE-F | GTGCGGAGGGGATGTGAAG | 140 |
| PCOLCE-R | CGAAGACTCGGAATGAGAGGG |  |
| COL7A1-F | TTACGCCGCTGACATTGTGTT | 93 |
| COL7A1-R | ACCAGCCCTTCGAGAAAGC |  |
| FBLN5-F | CTCACTGTTACCATTCTGGCTC | 89 |
| FBLN5-R | GACTGGCGATCCAGGTCAAAG |  |
| MMP13-F | ACTGAGAGGCTCCGAGAAATG | 103 |
| MMP13-R | GAACCCCGCATCTTGGCTT |  |
| MMP7-F | GAGTGAGCTACAGTGGGAACA | 158 |
| MMP7-R | CTATGACGCGGGAGTTTAACAT |  |
| MMP10-F | TGCTCTGCCTATCCTCTGAGT | 103 |
| MMP10-R | TCACATCCTTTTCGAGGTTGTAG |  |
| MMP1-F | AAATGCAGGAATTCTTTGGG | 200 |
| MMP1-R | ATGGTCCACATCTGCTCTTG |  |
| CEACAM6-F | TCAATGGGACGTTCCAGCAAT | 193 |
| CEACAM6-R | CACTCCAATCGTGATGCCGA |  |
| FUT3-F | CTGTCCCGCTGTTCAGAGATG | 131 |
| FUT3-R | AGGCGTGACTTAGGGTTGGA |  |
| CASP10-F | TAGGATTGGTCCCCAACAAGA | 210 |
| CASP10-R | GAGAAACCCTTTGTCGGGTGG |  |
| PTGS2-F | CTGGCGCTCAGCCATACAG | 94 |
| PTGS2-R | CGCACTTATACTGGTCAAATCCC |  |
| CXCL8-F | TTTTGCCAAGGAGTGCTAAAGA | 194 |
| CXCL8-R | AACCCTCTGCACCCAGTTTTC |  |
