# Supplementary material for: Heparanase Promotes Tumor Growth and Liver Metastasis of Colorectal Cancer Cells by Activating the p38/MMP1 Axis
Source: Front Oncol. 2019 Apr 2;9:216. doi: 10.3389/fonc.2019.00216 (PMC6454005; doi:10.3389/fonc.2019.00216)
Supplement: Supplementary file 7 [file Data_Sheet_1.docx]

Supplementary Material

Heparanase promotes tumor growth and liver metastasis of colorectal cancer cells by activating the p38/MMP1 axis

Xue Liu^†^, Zhi-hang Zhou^†^, Wen Li, Shi-kun Zhang, Jing Li, Ming-Ju Zhou, Jin-Wen Song^*^

*** Correspondence:** Jin-Wen Song: **songjinwenchina@yeah.net**


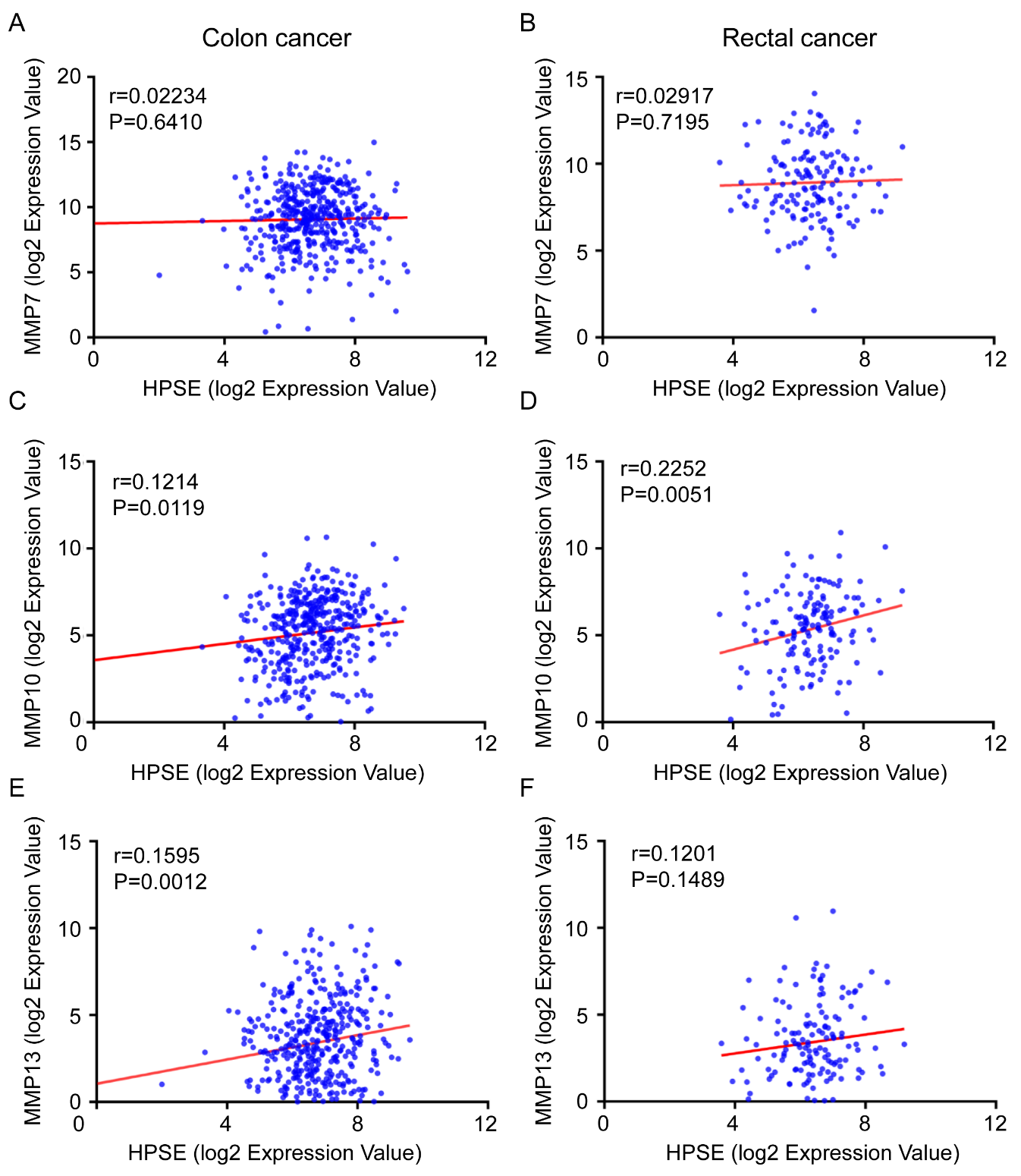


**Supplementary Figure S1. Correlation between HPSE expression and MMP7, MMP10, and MMP13 levels in colon cancer and rectal cancer.**

Correlation between the transcriptional levels of HPSE and (A, B) MMP7, (C, D) MMP10, and (E, F) MMP13 in colon cancer (left panel) as well as rectal cancer (right panel). The above analyses were performed using data from the Cancer Genome Atlas (TCGA). The correlation between gene expression levels was analyzed by the Person correlation test.
